# Participants’ perceived benefits from the GLA:D™ program for individuals living with hip and knee osteoarthritis: a qualitative study

**DOI:** 10.1186/s41687-024-00740-w

**Published:** 2024-06-26

**Authors:** Ania Kania-Richmond, Lauren A. Beaupre, Geneviève Jessiman-Perreault, Danika Tribo, Jason Martyn, David A. Hart, Jill Robert, Mel Slomp, C. Allyson Jones

**Affiliations:** 1 Bone and Joint Health Strategic Clinical Network, Alberta Health Services, Calgary, Alberta Canada; 2 Department of Community Health Sciences, Cumming School of Medicine, University of Calgary, Calgary, Alberta Canada; 3 Department of Physical Therapy, Faculty of Rehabilitation Medicine, University of Alberta, Edmonton, Alberta Canada; 4 Rockyview General Hospital, Alberta Health Services, Calgary, Alberta Canada; 5 Department of Surgery, Cumming School of Medicine, University of Calgary, Calgary, Alberta Canada

**Keywords:** Osteoarthritis, Lived experience, Exercise, Education, Management, Perception, Benefits

## Abstract

**Background:**

The **G**ood **L**ife with osteo**A**rthritis: **D**enmark (GLA:D™), an evidence-based education and exercise program designed for conservative management of knee and hip osteoarthritis (OA), has been shown to benefit participants by reducing pain, improving function, and quality of life. Standardized reporting in the GLA:D databases enabled the measurement of self-reported and performance-based outcomes. There is a paucity of qualitative research on the participants’ perceptions of this program, and it is important to understand whether participants’ perceptions of the benefits of the program align with reported quantitative findings.

**Methods:**

We conducted semi-structured telephone interviews with individuals who participated in the GLA:D program from January 2017 to December 2018 in Alberta, Canada. Data were analyzed using an interpretive description approach and thematic analysis to identify emergent themes and sub-themes associated with participants perceived benefits of the GLA:D program. We analyzed the data using NVivo Pro software. Member checking and bracketing were used to ensure the rigour of the analysis.

**Results:**

30 participants were interviewed (70% female, 57% rural, 73% knee OA). Most participants felt the program positively benefited them. Two themes emerged from the analysis: wellness and self-efficacy. Participants felt the program benefited their wellness, particularly with regard to pain relief, and improvements in mobility, strength, and overall well-being. Participants felt the program benefited them by promoting a sense of self-efficacy through improving the confidence to perform exercise and routine activities, as well as awareness, and motivation to manage their OA symptoms. Twenty percent of participants felt no benefits from the program due to experiencing increased pain and feeling their OA was too severe to participate.

**Discussion:**

The GLA:D program was viewed as beneficial to most participants, this study also identified factors (e.g., severe OA, extreme pain) as to why some participants did not experience meaningful improvements. Early intervention with the GLA:D program prior to individuals experiencing severe OA could help increase the number of participants who experience benefits from their participation.

**Conclusion:**

As the GLA:D program expands across jurisdictions, providers of the program may consider recruitment earlier in disease progression and targeting those with mild and moderate OA.

**Supplementary Information:**

The online version contains supplementary material available at 10.1186/s41687-024-00740-w.

## Background

Osteoarthritis (OA) is a degenerative joint condition associated with pain, functional limitations, and stiffness that impacts physical activity, work participation, mental health, and quality of life [1–5]. Evidence-based guidelines recommend treating OA using non-operative treatments such as education, exercise, and weight management as the first-line approach to managing hip and knee OA symptoms [6]. Despite the evidence supporting first-line approaches, studies consistently report a lack of uptake of these recommendations for a significant number of patients [7–9].

The Good Life with osteoArthritis: Denmark (GLA:D™) is an evidence-based program for symptomatic hip and knee OA that consists of 12 supervised neuromuscular group exercise classes held twice a week for 6-weeks, along with two structured education sessions [10–14]. The GLA:D program has been implemented in multiple countries including Australia, Austria, Canada, China, New Zealand, and Switzerland [15]. In Canada, as of 2022, the GLA:D program had been implemented in all 10 provinces and 2 of 3 territories [16].

Previous studies have demonstrated the GLA:D program’s effectiveness in reducing pain, improving quality of life, enhancing self-efficacy, and delaying joint replacement surgery among adults with moderate to severe hip or knee OA [12–15]. There has been limited work published on the qualitatively assessed experiences of individuals with hip and knee OA participating in the GLA:D program [17, 18]. Our previous work found the GLA:D program to be acceptable to participants and had a positive impact on a patient’s physical health routines and quality of life [19]. Ezzat et al. [18] found similarly positive experiences of the program in virtual as well as in-person delivery models.

Although patient-reported outcomes are captured using validated measurement tools, to our knowledge, no studies have focused on qualitatively exploring patient perceptions of the outcomes of the GLA:D program. Our study aims to fill this gap by examining how program participants perceived the benefits, or lack thereof, of the GLA:D program. Moreover, our study aims to supplement existing quantitative participant-reported outcomes from GLA:D Canada [20] to provide a contextual lens to the impact of GLA:D on daily routines, self-management approaches, and beliefs and attitudes towards exercise or physical activity. By pairing quantitative outcomes with the lived experiences of participants, we gain a richer understanding of the benefits, and potential drawbacks, of the program among people living with knee and/or hip OA. This contextual information is crucial to better understand and address potential barriers or challenges to implementation.

## Methods

### Study design

This study is a part of an overall evaluation of the province-wide implementation and spread of the GLA:D program in Alberta, Canada, informed by the RE-AIM framework [21]. This larger evaluation project had multiple objectives and companion papers focused on provider experiences in implementing the GLA:D program [22] and patient experience with the program [19]. We employed Thorne et al.’s [23] interpretive description approach to this qualitative inquiry. Reporting in this study is in alignment with the Consolidated Criteria for Reporting Qualitative Research (COREQ) checklist [24] (see Additional File 1). Ethical approval was obtained from the Health Research Ethics Review Board at the [blinded for review] (Pro00068308).

### Study setting

The evaluation was conducted in Alberta, Canada, the fourth largest province in Canada with a population of approximately 4.4 million, 50% of whom live in two metropolitan cities: Calgary and Edmonton [25]. Alberta has a single-payer public healthcare system which primarily covers physician-based services and hospital-based care. Limited public funding is available for rehabilitation services (e.g., physical therapy, occupational therapy); however, private rehabilitation services are also available [26].

### Participant selection

We employed a purposive sampling strategy to generate a diverse sample of individuals who participated in the GLA:D program. The sampling aimed to maximize variation across geography, clinical settings, and gender. Participants were included if they met three criteria: (1) aged 18 years or older, (2) living with symptomatic hip or knee OA, and (3) attended at least one of the GLA:D program sessions between January 2017 and December 2018 in Alberta. Recruitment occurred in nine clinics from the initial cohort of 12 clinics that implemented the GLA:D program in 2017. These clinics included both public healthcare centers and private clinical settings that were located in rural (n = 17) and urban or metropolitan areas (n = 13) [27]. From these clinics, 96 participants consented to be contacted. Of those who consented to be contacted, 12 provided incorrect contact information, 51 were unable to be contacted after attempts, and 33 participants were successfully contacted.

### Data collection

We conducted semi-structured telephone interviews with participants, on average, four months (ranging from 1 month to 12 months) after completion of the GLA:D program. Informed consent was obtained from each participant prior to the interview. Interviews were guided by a semi-structured interview guide (see Additional File 2). Data collection ended when no new insights were generated through subsequent interviews, as determined by consensus during the analysis process (i.e., data saturation) [28].

### Data analysis

All interviews were conducted by two research team members (AKR and EM) and were audio recorded, transcribed verbatim, and de-identified for analysis. NVivo Pro12 software was used to support data management and the analytic process. Interviews lasted between 20 to 60 minutes. A thematic analysis approach was employed, which was initiated with the development of descriptive codes and categories. Two researchers (DT and AKR) independently conducted initial data coding and established agreement on code categories and data interpretation. Descriptive analysis was followed by an interpretive analysis, where the researchers clustered and re-clustered descriptive categories to inductively identify emergent themes and sub-themes. Emergent themes were validated by a third researcher and reviewed by three research team members (LB, AJ, and GJP) to confirm logical presentation and alignment with the study objective. To enhance the quality of the analytic output, a code-recode strategy was used whereby coders undertook repetitive analyses of data segments, comparing their own coding for consistency, and further refinement of emergent categories. Regular meetings were held to discuss the emerging findings and personal reflections that enabled team members to unpack their potential biases and perspectives about the findings. A description of the research team members’ backgrounds is provided in Additional File 3.

## Results

Thirty participants completed the interview. Participant characteristics are presented in Table 1. The majority of those interviewed were female (70%), living in rural settings (57%), participating in a publicly covered GLA:D program (80%), living with knee OA (73%), and participated in physical activity before beginning the GLA:D program (63%). Regarding program attendance, 60% of our sample reported completing the program without missing any classes and 17% did not complete the program.

**Table 1:** Description of study and program participants (n = 30)

Participant and program descriptors	Frequency	Percent
Sex		
Female	21	70%
Male	9	30%
Participant location		
Urban setting or metro setting *(population of 25,000 or more)*	13	43%
Rural setting *(population less than 25,000)*	17	57%
GLA:D payment model		
Attended a publicly paid program	24	80%
Attended a privately paid program	6	20%
Type of OA		
Attended GLA:D for hip OA	8	27%
Attended GLA:D for knee OA	22	73%
Physical activity participation		
Participated in physical activity prior to GLA:D	19	63%
Did not participate in physical activity prior to GLA:D	11	37%
Program attendance		
Completed the program, no missed classes	18	60%
Completed the program, but missed classes	7	23%
Did not complete the program	5	17%

## Themes

Most participants (77%) described some level of benefit from their participation in the GLA:D program. Approximately a quarter of the participants (23%) perceived no benefits at all from their participation due to persistent and/or exacerbated pain and their perceived severity of OA. Of those who found the program beneficial, less than one-third (27%) only perceived limited benefits from the GLA:D program, citing reasons such as a failure to meet their anticipated goals and a lack of overall improvement. Two themes emerged that encapsulate the perceived benefits of GLA:D from the participant’s point of view: Wellness and Self-Efficacy.

### Wellness

The majority of participants (77%) identified that the GLA:D program had a positive impact on their health and well-being, albeit to varying degrees. Three emergent categories illustrated the improved wellness experienced by participants: (1) pain reduction and management, (2) improved mobility, and (3) improved strength. Selected quotes are referenced in this section, with the remaining quotes presented in Table 2.

**Table 2:** Selected quotes on the theme of wellness and the sub-themes of pain reduction and management, improved mobility, and improved strength

Sub-theme	Quote number	Quote
Pain reduction and management	1	“…my knee doesn’t hurt.” (Participant 20)
	2	…I’ve had issues with it come up, but I know what to do. And it has not given me that kind of pain.” (Participant 20)
	3	“…yeah I went, because of my knee for sure… it improved, my knee. If it was sore and I did the exercises… it seemed like the exercises would take the soreness out of my knee.” (Participant 21)
	4	“… my knees have reduced in pain, and that’s partly because… I continued at least some of the exercises, if not all. (Participant 2)
	5	“…when I stop doing those exercises, my knee acts up… it’s hurting more. Yeah. And I know it’s ‘cause I’ve got to get back to it.” (Participant 1)
	6	“I do notice when I don’t do them for a length of time that my knees start to get sore again.” (Participant 5)
	7	“…it was great because we did the exercises and I felt better. If I remember—but I’m still trying to do exercises. I continue it… I have less pain in the left—in the right leg. And as I had before. And that was my weakest leg.” (Participant 8)
	8	“… it didn’t cure it. But I’m beginning to feel now that… my knee doesn’t bother me much… my calf is, is not as—doesn’t feel like it’s going to break up… it was sore… and my hip… has improved with time.” (Participant 8)
	9	“… now that my knee is not hurting as bad, I still do some of the exercises… that I was given.” (Participant 27)
	10	“…at the beginning, well, T3 is my friend. [laughs] Yeah. And I do drive a standard so I would have to say the first couple weeks there, driving home, it was hard to clutch, because it’s my left knee. I’m like, oh my God, what have I gotten myself into?” (Participant 1)
	11	“I guess if it had been hurting I would have noticed. But it wasn’t, so I just continue on.” (Participant 1)
	12	“I thought that I was probably, probably in more discomfort than I was when… I started it. I had pain at night especially… I was taking a lot of pain medication to get to sleep because my knees ached at night.” (Participant 28)
	13	“I think the timing of it, the program probably extended my muscle pain, just because I never gave it a rest. And then I quit. They sent me for physio and they sent me—I went for massage and for chiropractic and nothing helped. Finally, I quit everything and by June I was good again. It healed up on its own, when I left it alone…” (Participant 18)
	14	“We did test results, see how we stand up on certain things she had us do the first time. And in every one, I was better than I was at first. But I was having more pain. That was sort of the irreconcilable fact that although I was performing better, I was having more pain.” (Participant 28)
	15	“The only part that was missing for me is, they kind of made this program like, well, if you exercise your pain’s going to go away… maybe there’s a few people, but most of the people were well over 40, that were in this program. And the pain is never going to go away for them, so it, it kind of left you with, okay, now I’ve got to go find someone else to help me.” (Participant 32)
	16	“I really hurt myself doing some of the exercises at, at GLAD. And [provider] was really, really compassionate. And, and tried to adapt it for me, but I was kind of scared, just to even push myself a bit… I kind of did a very modified program and I watched the other people progress.” (Participant 11)
Improved mobility	17	“…I have arthritis and I thought it might help with my disabilities… I came out of the program with a lot more… I’ve learned, like to, instead of just leaning on the table or an arm of a chair to get up off of a chair, or even, or when you’re sitting on a bathroom, the toilet, to get up, rather than hanging into something. And I found when I go up and down the stairs I found too, my knees might wobble, but so they’re straight… I found out a lot of, I guess, different things that I was searching for… I know my body is really out of shape. But there’s certain things I can’t do because of my problems.” (Participant 3)
	18	“I do walk… that had been one of the goals that I wanted to be able to walk longer distances again, and cross country ski. And we were, [partner] and I were able to do that last year. Three times, anyway. So that was good.” (Participant 15)
	19	“…about four weeks into the exercises, I found that I could get on the horse. But what really surprised me was, it was not my knee that improved as much as my hips. Where I, when I swung onto the horse prior to that I had trouble getting my leg over the cantle. And when I swung on I looked down and I cleared it by at least six inches.” (Participant 21)
	20	“I’m more mobile than I was. Like I seem to be able to get around a little bit more.” (Participant 24)
	21	“Like even just simple things like going up stairs, right? If I… lock it in my head that, hey, use your gluts…Yeah it’s a totally different kind of feeling than when I, when I don’t do that, right? I find that I don’t have as much trouble with that (stairs) any more as I used to…” (Participant 5)
	22	“It has helped me in my daily life, in terms of handing stairs, and handling other weightlifting…not in terms of weights, but in terms of, well, just handling suitcase and so on, which we must in traveling… so it has, it has been a significant benefit to me.” (Participant 16)
	23	“I was able to do the stairs better after, after that program.. And getting off the toilet was another one.” (Participant 17)
	24	“I’ve gained so much mobility. Like I can roll over in bed now without any pain hardly. Where I couldn’t before… this has made my life easy, you know—easier.” (Participant 22)
	25	“…just a simple thing. But I was walking wrong and it was causing more problems with my sore ankles. Which was probably causing problems for my knee and my hip. And it’s a simple thing, how to walk properly… (Participant 20)
	26	“I’m quite active, and my wife always told me to stop limping. And the part of limping is I think, you’ve been doing it for so long that it becomes a habit. And so I’ve really been concentrating on, if it doesn’t hurt there’s no point in limping.” (Participant 21)
	27	“… I have a big yard, big garden, so I garden… I could actually do my gardening much easier last summer. And then this summer again too.” (Participant 12)
	28	“…even just if I think about doing housework… when I first started having pain, like more extensive pain and stuff, it was very difficult for me to do any type of housework. Or I’d have to sit down, you know, after like 15 minutes and rest or whatever, right? But now I’m able to do, you know—I can extend that for a lot longer.” (Participant 5)
	29	“I went to BC for my niece’s grad and she said, Auntie, you know what? You are faster than you used to? I said, really? She said, yeah. And you’re not limping. I said, oh I didn’t even notice. It was… because I was doing it so much. I didn’t notice it. And I guess because I wasn’t in pain, I just kind of went along with life.” (Participant 1)
	30	“…I was fencing one day and uh, I had to cross the fence and for the first time in years I swung my leg over the fence instead of crawling through the wires. So it was quite remarkable for me.” (Participant 21)
	31	“I skied 55 times cross country skiing over the winter. I still go to the gym two, three times a week… I’m biking… I can hike five kilometers, no problem now. So it’s made a world of difference.” (Participant 13)
	32	“It’s great, I loved it. I think it, I went to my first Farmers’ Market this summer. And I haven’t gone to the Farmers’ Market in about five or six years. Because…I knew I couldn’t walk it. But I went to a Farmers’ Market and we hung out for two hours and walked around. I got in the car, I said, I’m tired, but—wow! That was awesome because… there’s so many things that I stopped doing because I was in so much pain. And now I just do stuff.” (Participant 1)
	33	“I loved it. I think everybody should do it… I’m a big promoter of the program, absolutely. Because I’m at a level right now where I’m thinking, I don’t need surgery! Seriously.” (Participant 1)
Improved strength	34	“I don’t recall it helping the pain in my knee any—because my knee was worn out on that one side. But I don’t recall of any difference in the pain. But the exercise, it definitely helped my, my leg strength.” (Participant 25)
	35	“…I think I was much stronger. My legs were stronger. And last summer I did do some hiking, which I had not done the summer before”. (Participant 12)
	36	“…my legs got stronger. They were getting weak… with the strength in my thighs, with the exercises I was doing, I felt a lot more confident and was able to push a little harder. And you know, few times pushed too hard, but you know that so then you just backed off… I could also think about doing other exercises, like some other stretching or doing a little bit of yoga or something like that, that I haven’t done for years. So yeah I felt a lot better.”(Participant 29)
	37	“All in all, I think that they have benefitted both my stability and, and encouraged the more development of strength in my muscles.” (Participant 2)
	38	“…I probably would have paid if there had been a spot because you know, I know my legs were stronger… we moved into a condo building and there is an elevator, but generally I use the stairs. And, and I was able to do the stairs better after, after that program.” (Participant 17)
	39	“Because really the exercises are, are not really grinding it, I don’t think. They’re strengthening everything around it. To hold it so it doesn’t grind so bad.” (Participant 1)
	40	“… I thought that I could, you know, learn more about how, how to strengthen my, you know, other areas of my body so that they would carry the load differently. And it would put less stress on my knees, to avoid, you know, surgeries and that kind of thing.” (Participant 4)
	41	“When you can walk a little bit better, when you can see… that you can strengthen your knee, psychologically that has to be a plus.” (Participant 9)
	42	“I think it was the exercises would strengthen the muscles and that would help you take the stress off the joints. I think that was the key—so I clicked into that right away…So there’s always something I can do. Strengthen my gluts or the hip abductor muscles or what not. But I think, you have to buy into the program. It’s a lifestyle change.” (Participant 13)
	43	“Well I can tell you that even during the program itself I noticed increased strength in my knees specifically. And it increased my motivation to actually do more to actually strengthen the muscles in the knee, both the side muscles as well as the front and back muscles in my legs and knees.” (Participant 16)
	44	“I can see where they’re geared towards strengthening. We worked on knees, so strengthening the knee muscles, which was always a concern of mine, which—I was always thinking, which exercises do I need to do to really keep the muscles and tendons and ligaments strong, right? So here was my answer, right? The GLAD program. But again, this happened, and so I took advantage of it as much as I could.” (Participant 18)
	45	“Uh yeah. I, my uh, my legs got stronger. They were getting weak. And um so with the strength in my thighs, with the exercises I was doing, I felt a lot more confident and was able to uh, push a little harder.” (Participant 29)
	46	“And I think what happened is the GLAD stuff targeted much more and they actually, I’m probably jumping ahead. But my, they helped my muscles strengthen a lot.” (Participant 31)

#### Pain reduction and management

For some participants, the GLA:D program was an effective way of addressing their OA-associated joint pain. Over one-third (37%) of the participants reported pain reduction and three participants even reported being pain-free after completing the program. Many recognized that they needed to continue with the GLA:D exercises after program completion to effectively continue to manage their pain; the exercises provided a mechanism by which to manage their pain more effectively and independently. The relationship between the exercises and pain is reflected in the experience of Participant 21:

…… it improved, my knee. If it was sore and I did the exercises… it seemed like the exercises would take the soreness out of my knee. (Participant 21)

The connection between proper joint alignment and pain was also made, resulting in a greater focus on quality of movement. As explained by Participant 5:

…that whole idea of the mechanics of, of your movements. That kind of helped me,… if I’m finding that I have pain on a set of stairs, if I go and do it the next time, thinking about you know, engaging my glutes, my core and that type of thing, right? (Participant 5)

Exercise as a pain management strategy was a new understanding, particularly for those who generally avoided activity, helping to resolve a fear that activity would cause more harm to their affected joint.

Program participation increased pain levels and caused significant discomfort for almost one-quarter of participants (23%) many of whom self-reported that they have more advanced OA. One-third of these participants felt the increased pain was too much to continue and opted to discontinue the program. Some of those who continued through the program, despite the initial pain, reported functional improvements (e.g., lifting, walking, stairs, returning to activities) from exercising and, later, a reduction in pain. For others who progressed through the program, this was not the case and they experienced persistent pain throughout, as stated by Participant 28,

We did test results, see how we stand up on certain things she had us do the first time. And in every one, I was better than I was at first. But I was having more pain. That was sort of the irreconcilable fact that although I was performing better, I was having more pain. (Participant 28)

#### Improved mobility

Descriptors such as *stamina, stability, speed, improved joint alignment* and *range of motion*, particularly with knee extension underlie the perceptions around improvements in mobility. From the participants’ perspective, these physical gains also translated to ease of movement and functional improvements, which impacted their overall sense of well-being. Almost half of the participants (43%) reported that daily life activities were no longer difficult: using the bathroom, going up and down stairs, turning in bed, gardening, housework, regular exercise, and playing with grandchildren on the floor; all became possible with their improved mobility, as reflected in the experiences of Participant 17,

I was able to do the stairs better after, after that program.. And getting off the toilet was another one. (Participant 17)

With improved functioning and mobility of the joint, over half of the participants (53%) became more physically active, beyond the exercises delivered through the program. Participants caught themselves re-starting activities they had stopped because the pain and poor mobility were no longer limiting factors; for example, engaging in more intensive physical activity such as biking, hiking, horseback riding, curling or cross-country skiing. As reflected in the experience of Participant 21:

…about four weeks into the exercises, I found that I could get on the horse. But what really surprised me was, it was not my knee that improved as much as my hips…when I swung onto the horse prior to that I had trouble getting my leg over the cantle. And when I swung on I looked down and I cleared it by at least six inches. (Participant 21)

#### Improved strength

Almost half of the participants (47%) felt muscle strengthening was an important benefit. Some participants indicated they had prior knowledge about the benefits of strengthening exercises to manage their OA, while most acquired this new knowledge throughout the program. They felt the program offered strengthening exercises that targeted the joint specifically. Some participants also realized that improved strength stabilized and reduced stress on their joints, contributing to pain management, and improved mobility. Participants expressed that these strength gains allowed them to engage in activities that took advantage of, and helped maintain, that improved strength, as stated by Participant 12,

…I think I was much stronger. My legs were stronger. And last summer I did do some hiking, which I had not done the summer before. (Participant 12)

In addition to the above, participants described indirect wellness benefits resulting from their participation in the program such as changes in their overall physique due to weight loss and overall muscle toning from regular engagement in physical exercise. Overall, the benefits and gains from the program met, and at times exceeded, many participants’ expectations (53%); one participant expressed that given the improvements experienced, joint replacement surgery was no longer a consideration for them,

I loved it. I think everybody should do it… I’m a big promoter of the program, absolutely. Because I’m at a level right now where I’m thinking, I don’t need surgery! (Participant 1)

### Self-efficacy

Participants indicated that the program’s structure, group format, weekly commitment, and exercise progressions left them feeling empowered to better manage their condition. Three emergent sub-themes characterized the self-efficacy experienced by participants: (1) confidence regarding physical activity and exercise, (2) awareness of movement, and (3) motivation to be and remain active. Selected illustrative quotes of these sub-themes are presented in text with additional quotes provided in Table 3.

**Table 3:** Selected quotes on the theme of self-efficacy and sub-themes confidence, awareness, motivation

Sub-theme	Quote number	Quote
Confidence	1	“But it was also presented that…the proper exercise does not make it worse. So now I don’t have to worry about my walking and stairs… it should stabilize or get better… that was the biggest thing.” (Participant 18)
	2	“Yes well it’s more my confidence in my knees that improved.. Well I went out this morning and walked—I had a counter on. I did 4,000-something this morning… I can go out on an average day between 3,000 and 4,000. But I take a walking stick.” (Participant 14)
	3	“Oh I think the program was a good program… it did help me and give me a little more confidence because I was worried about it…” (Participant 25)
	4	“Well I never considered getting on… an exercise bike. I can’t do the upright bike but I found out I can actually go on the recumbent bike with hip… But that bike, I found—I would have never done that on my own…” (Participant 24)
	5	“…with the strength in my thighs, with the exercises I was doing, I felt a lot more confident and was able to push a little harder. And you know, few times pushed too hard, but you know that so then you just backed off.” (Participant 29)
Awareness	6	“I notice um, we have quite a few stairs—to come into the house we have steps you need to come down… I’m very conscious of keeping my feet straight and… to some extend I can feel like I can tighten… my thigh muscles…and my calf muscles to keep my knee above my feet as well.” (Participant 15)
	7	“A little bit… I’m still retraining my brain. How to walk without a limp. And that’s the hardest part… but when I walk without the limp there’s hardly any pain. But when I lose control over the brain. and I start limping—I’ll get um really bad pain.” (Participant 22)
	8	“Yeah like I couldn’t walk… hardly at all without having pain. So it, I basically gave up on the walking. But now, I can walk if I focus I can walk without too much pain. If I’m focused.” (Participant 22)
	9	“Like even just simple things like going up stairs, right?…if I lock it in my head that, hey, use your gluts… Yeah it’s a totally different kind of feeling than… when I don’t do that, right? And even to this day… if I’m finding that I have pain on a set of stairs, if I go and do it the next time, thinking about you know, engaging my, my gluts, my core and that type of thing, right?…not putting the load, like not overextending my knees so that the load of my weight is you know, being, my knee is taking the brunt of it…” (Participant 5)
	10	“I have my, my exercises kind of set up in my basement. And I… have it all there. I have been not entirely 100 percent dedicated to doing them every, every couple of days… when I do do them… I do feel better… I have it at home and… over the summer, I’ve kind of not done as much. But I have to get back on track with that.” (Participant 5)
	11	“…when I stop doing those exercises, my knee acts up… it’s hurting more. Yeah. And I know it’s ‘cause I’ve got to get back to it.” (Participant 1)
	12	“I do notice when I don’t do them for a length of time that my knees start to get sore again.” (Participant 5)
	13	“I also realized though, because I was taking aquacise regularly, and I learned through… the physiotherapist and through the GLAD program that I was doing some of the exercises wrong in aquacise, which was causing more issues with my knee…” (Participant 20)
Motivation	14	“it (GLA:D) is quite simple. That’s the key to it. If you get a whole lot of complicated exercises, you say you don’t have time to do it… I can do stuff that they had in about ten minutes. As long as you do it every day…the surgeon I had..said the only thing to do is to keep moving. Just keep moving, which is what the GLAD program says.” (Participant 14)
	15	“But, perseverance. I never stopped. There was even—there was even one day… I’d had nothing to do with my knee. I hurt my back. And I went and I almost didn’t go because my back was so bad. And I was doing the exercises. And I was crying through. And [provider’s name] was like, are you okay? Yup… it’s my back. It’s not my knee. I’ll just keep going. It’s okay. Because I, you know, just do it! Because it makes it better.” (Participant 1)
	16	“…They were all good and I really enjoyed the program. I was actually charged up about it. It turned me on… when you do that and you take that, what you learned, and take it to the gym… it’s a lifestyle change. You have to keep working out and keep on the program… if I don’t go to the gym a couple weeks or something I get, I’d better get at it. And go back. And that happens. You don’t want to lose it.” (Participant 13)
	17	“Well I can tell you that even during the program itself I noticed… increased strength… in my knees specifically.. it increased my motivation to actually do more to actually strengthen the muscles in the knee, both the side muscles as well as the front and back muscles in my legs and knees. So I think that the GLAD program increased my motivation to do specific things, because until then I didn’t have as much knowledge as to what would be best, or what would, what would work best.” (Participant 16)
	18	“…it pushed me to do more than what I would have. I could easily have been lazy and… just have gotten to… a comfortable point. Whereas um this one pushed me. And I have to admit, I needed that. It was great…” (Participant 29)
	19	“The exercises were a little bit different… a lot of similarities… physiotherapy gave us things that we could do at home… the GLAD program had us actually in the room with the exercise equipment… that would allow for more specific and direct involvement with the exercises. I found them all useful… what I think some of the some of the lecture did, was help to understand why certain exercises were included, in terms of working on particular muscles. Or, ligaments, or well, specifically muscles. And, and alignment of joints, and why. And so on. So I found… that they all had their, their importance.” (Participant 2)
	20	“I could also think about doing other exercises, like some other stretching or doing a little bit of yoga or something like that, that haven’t done for years. So yeah I felt a lot better”. (Participant 29)

#### Confidence

Participants left the program with a stronger sense of confidence in managing their condition, which was closely linked to learning. Almost half of participants (46%) felt the program served as an opportunity to review and build upon their existing knowledge; however, for the majority of participants (53%), the program offered new knowledge and insights about how to effectively manage their OA. As expressed by Participant 20:

… just a simple thing… I was walking wrong and it was causing more problems with my sore ankles. Which was probably causing problems for my knee and my hip. And it’s a simple thing, how to walk properly. (Participant 20)

Enhanced confidence also addressed underlying uncertainty regarding movement and what to do with sensations experienced by participants during movement that often resulted in hesitation and, at times, ceasing activity altogether. In addition to a better understanding of correct movement, experiencing improvements in strength and mobility directly impacted participant’s certainty and motivation to engage in physical activity. For example,

…it’s more my confidence in my knees that improved… I can go out on an average day between 3,000 and 4,000 [steps]. But I take a walking stick. (Participant 14)

#### Awareness

For over one-third of participants (37%), an important benefit of the program was the development of awareness– joint alignment, body position, and correct movement techniques. This awareness was applied when exercising and during functional activities such as sitting down, standing up, and walking. As explained by Participant 22,

A little bit… I’m still retraining my brain. How to walk without a limp. And that’s the hardest part… but when I walk without the limp there’s hardly any pain. But when I lose control over the brain… and I start limping—I’ll get really bad pain. (Participant 22)

Beyond an understanding and application of proper alignment in movements, participants described a better awareness of the connection between pain and exercise and how they used this understanding to maintain or increase their physical activity and mobility. For example, some noted that increased mobility reduced pain and that increased pain signalled a need for more mobility (and movement). As stated by Participant 1:

…when I stop doing those exercises, my knee acts up… it’s hurting more. Yeah. And I know it’s ‘cause I’ve got to get back to it. (Participant 1)

Such awareness resulted in an important shift in the understanding of OA– whereas, before the GLA:D program, pain was often a debilitating factor that prevented movement, afterwards the sensation of pain was used by participants as an indicator they needed to resume exercise or practice specific exercises once more.

#### Motivation

The GLA:D program appeared to be a source of motivation for participants to exercise and to remain physically active. For over half of the participants (53%), the program was the vehicle by which regular exercise became an established or re-established part of their daily routines. For some, it provided the structure that enabled perseverance, particularly through the initial stages of the program which had challenges associated with learning new exercises, overcoming de-conditioning, and a lower fitness level. For example, Participant 29 stated,

…it pushed me to do more than what I would have. I could easily have been lazy and… just have gotten to… a comfortable point. Whereas this one pushed me. And I have to admit, I needed that. It was great.. (Participant 29)

For others, the benefits experienced throughout the program (e.g., the health improvements, newfound confidence in their physical abilities, independence to exercise at home, and direct link between exercise and pain) were motivating factors to continue with the program and maintaining an exercise routine after program completion. As stated by Participant 16,

Well, I can tell you that even during the program itself I noticed… increased strength… in my knees specifically.. it increased my motivation to actually do more to actually strengthen the muscles in the knee, both the side muscles as well as the front and back muscles in my legs and knees. (Participant 16)

**Fig. 1:**
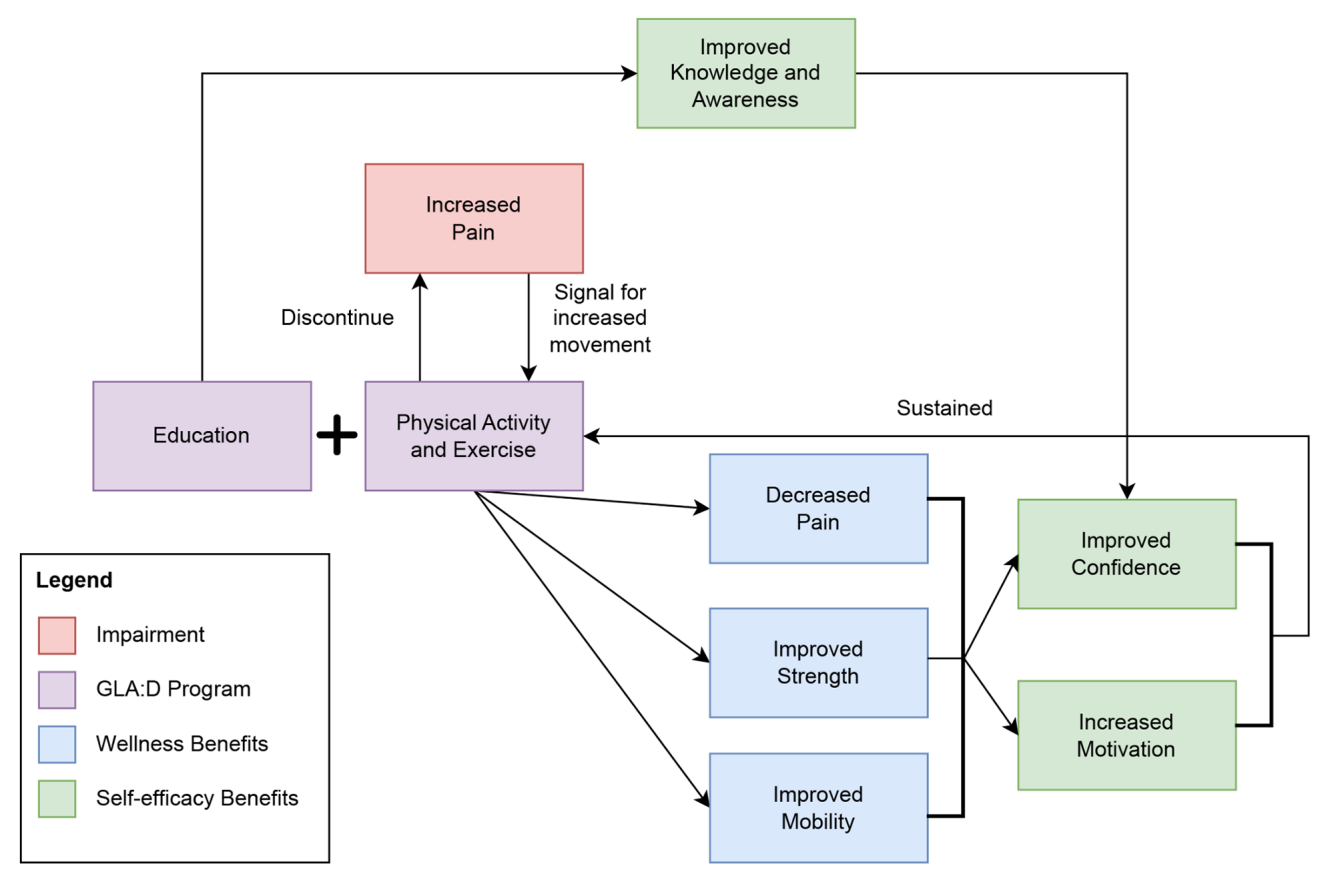
Conceptual diagram of the connection between wellness and self-efficacy themes and sub-themes

Overall, the analysis of participant interview data resulted in several sub-themes on the benefits of the GLA:D program, many of which align with the quantitative measures reported in the GLA:D annual reports [20]. Yet, these quantitative measures are not independent of one another; instead, they are interrelated constructs, and these connections were elucidated through the participants’ perceptions and experiences. Figure 1 below visualizes the connection between the sub-themes.

Participants’ experiences suggest that pain had a bidirectional relationship with movement and exercise, whereby increased mobility through the exercises in the program led to reduced pain. As the participant decreased their movement, an increase in pain signalled the need for more movement and exercise. This realization was a result of increased awareness, gained throughout the program which helped the individual redefine pain as not a debilitating factor but an indicator to promote self-management. The outcomes of physical activity (i.e., increased mobility and strength) were related to awareness, confidence, and motivation. For example, participants gained familiarity and confidence with the exercises as well as an awareness of their body’s alignment while performing those exercises. This allowed individuals to become comfortable exercising again and the benefits gained during the program motivated individuals to sustain physical activity after program completion, thus supporting sustained behaviour change and self-management of their OA.

## Lack of benefit

The program was not perceived as beneficial for all participants in managing their OA-associated symptoms (see Table 4 for supportive quotes); 23% of participants felt they did not benefit at all from the program, due to experiencing minimal or inconsistent gains in pain reduction and/or improved mobility. Two participants also noted other health issues that precluded effective participation in the program and hence did not benefit from the program. The majority of those who did not feel they benefitted from the program felt their OA progressed too far for them to be good candidates for the program. For example, one participant stated,

I was too far gone for the GLA:D program. (Participant 11)

**Table 4:** Selected quotes on the lack of or limited benefits

Quote number	Quote
1	“I can’t do that half of the exercise. Or things like you know, I, I couldn’t do any of the, any of the things on the floor because once I’m on the floor I cannot get up… it was difficult… at the end of the first session I went home and then later that evening I started having muscle spasms… So I withdrew myself from the program because um, yeah the muscle spasms are just not a place I want to go.” (Participant 4)
2	“Walking down stairs is one of the hardest and scariest things I have to do. I’ve been going sideways down stairs for about six years now. I can’t walk normally down stairs, like people do. And that was my one thing that I said, I want to walk stairs. And I can’t, I can’t do stairs now.” (Participant 1)
3	“And I didn’t really totally complete the program. I did go back and sort of, we adapted it a bit and everything…I needed the knee replacement and you know, not everybody does…I was too far gone for the GLA:D program.” (Participant 11)
4	“…my knee was so bad already that I didn’t see that much improvement. I knew it got stronger. But yeah it depends on what stage you’re at, I think….I do hope more people can benefit from it. And that people will take it before they get to the point where there’s no return. You know what I mean? Before it, there’s so much damage.” (Participant 17)
5	“… she (the GLAD provider) said to me on the last day, she said, you know, [participant name] you might have just had been better off if you’d gotten into the program earlier. You might have had a better chance for success. And I said, well, I sure wish I had known about it earlier… that would have made a difference.” (Participant 28)
6	“There were people that, like I said, were in a lot of pain, waiting for you know, knee replacements or things like that… they really couldn’t take advantage of the program, although it did help some… when you’re in a lot of pain it’s pretty hard to follow through with every movement… kind of curtails your, your ability to… be able to get some product from, from the exercises.” (Participant 9)
7	“But if I’d maybe known that I… was not the best candidate, maybe it’s like every medical procedure—no results are guaranteed when you do what you do, see how it works out.” (Participant 28)
8	“…but the bottom line was that I didn’t think that I had improved that much…I just kind of gave up because I didn’t think it was helping me. And at the same time, I was using a cane…and sometimes during the day the pain was not bad…. And you know, I could get around and everything anyway…” (Participant 28)
9	“…hard to say that it actually made any difference at, at all… sometimes you know, it would be better, sometimes it, it wouldn’t…I still had a hard time why it wasn’t getting better because of doing the exercises.” (Participant 19)
10	“No I don’t think I noticed really any changes. I mean, the whole thing was new to me because I don’t exercise. And I was kind of glad to learn the exercises. But then afterwards I never bothered doing any more because I didn’t see any—anything happening from the exercise.” (Participant 30)
11	“The assessment afterwards showed improvement. It’s a condition that doesn’t improve as such… my activity level, I guess, was, was better. Um but it doesn’t change the underlying physiology of the situation. So I still have problems with my knees…but it did help in the short term, for sure.” (Participant 10)
12	“…my knee was so bad already that I didn’t see that much improvement. I knew it got stronger….So it (the benefit) was pronounced as far as strength goes…but pain—possibly but definitely not as pronounced…There’s just some things you can’t overcome.” (Participant 17)
13	“I don’t recall it helping the pain in my knee any—because my knee was worn out on that one side. But I don’t recall of any difference in the pain. But the exercise, it definitely helped my, my leg strength.” (Participant 25)

These participants reflected on the need for GLA:D to be available earlier in their disease progression to manage expectations in a more effective way for those with very advanced OA, as stated by Participant 28,

… she [the GLA:D provider] said to me on the last day…you might have just had been better off if you’d gotten into the program earlier. You might have had a better chance for success. (Participant 28)

Over one-quarter of the participants (27%) had a mixed response to the program, whereby they described it as providing benefit to some but not all aspects of their OA-associated experiences. For example, some found it reduced their pain levels, but they were unable to achieve their anticipated goals (e.g., walking down the stairs confidently). Others recognized improved strength, but they did not experience consistent pain reduction. Lastly, as noted in the above section on wellness, some experienced persistent pain throughout the program, which they associated with the program exercises.

## Discussion

This qualitative interpretive description study examined participants’ perceived benefits experienced through their participation in the GLA:D program in Alberta, Canada. Most participants felt they benefitted from the program, which led to improvements in mobility, activities of daily living, and pain management, as well as increased their motivation, awareness, and confidence in managing pain and becoming more physical active. However, almost one-quarter of participants did not perceive the program to be beneficial. Lack of mobility and/or unrelenting joint pain were reasons why they felt their OA was too advanced to benefit from the program. The qualitatively described benefits, and lack thereof, reported in these interviews provide added depth to the quantitative outcomes reported in the GLA:D Annual Report [20] and helped to elucidate the connections between the different types of benefits.

We found several program benefits reported by participants were not currently being measured in the GLA:D database but were important to participants. First, participants also described their growing confidence to exercise, engage in activities of daily living, and manage their OA symptoms. This confidence represents a sense of self-efficacy and is an important construct in many health behaviour change models [29–31]. Self-efficacy is associated with adherence to exercise regimes among individuals living with OA [32, 33], therefore, the growth of self-efficacy as a part of GLA:D can help sustain its benefits after program completion. Second, ancillary benefits such as improvement in strength were frequently mentioned. These benefits were associated with participation in the program exercises and acted as a motivating factor to continue to practice the GLA:D exercises or other physical activity which thus resulted in increased strength. This strengthening was described in reference to the areas targeted by the program (e.g., legs, glutes) as well as other muscle groups indirectly targeted by the program exercises (e.g., abs, arms). This finding enhances our understanding of the benefits of the programs beyond the information on functional movements tests (i.e., 30-second sit stand and 40-metre walk test) and sport and recreation-related physical function that are captured in the GLA:D database. Moreover, this strengthening was closely tied with an important success factor for many participants- their ability to return to previous activities that had become out of reach due to their OA.

Findings from this diverse sample of participants indicated a variety of views on the benefits of the GLA:D program. Many of the participants interviewed reported better pain management. Yet not all participants experienced pain relief; some participants in the present study indicated that participation in the program increased their pain. These contrasting experiences of movement and pain may help to explain the findings of a quantitative study which found that both improved and worsening pain were associated with physical activity among GLA:D program participants [34]. Some participants experienced immediate pain after the first session and were not able to continue. This response to the exercises is expected from some participants and providers are trained to prepare participants for this initial pain reaction and reassure them that the pain will likely subside over time. These perspectives highlight a need to better support participants in this initial stage of the program. Past research examining gender-related differences in pain responses found that program-related factors such as attending former participant lectures and attending more exercise classes impacted women’s likelihood of experiencing pain reductions while men’s mental health and comorbidities impact their likelihood of experiencing pain reductions [35]. Based on these responses and findings from others, the program may need a tailored approach informed by factors such as gender. Future research should focus on examining what factors impact the likelihood of obtaining clinically relevant pain relief.

The other participants who continued in the program, but experienced worsened pain described themselves as poorly suited to the program. These insights highlight the need to provide people living with knee or hip OA with an intervention through referrals to the GLA:D program in the earlier stages of OA. Given that these study results represent findings from some of the first individuals to participate in the GLA:D program in Alberta, it is possible that individuals with knee and hip OA were referred regardless of OA severity. This is supported by findings from our previous evaluation of GLA:D providers who stated that they often struggled with recruitment [22]. These novel findings on GLA:D participants’ experiences with pain may elucidate some reasons as to why a subset of participants are not experiencing pain-related benefits but experiencing increasing pain, a trend observed in Canada, Australia, and Denmark [11]. As the number of sites offering the GLA:D program grows and awareness of the program increases, these findings provide important insights that can assist in continued quality improvement of global implementation of GLA:D.

This study has several strengths. First, to our knowledge, this is the first study to report participants’ perceptions of the benefits of the GLA:D program, providing deeper insights into the program benefits, some confirmatory and some novel, experienced by participants. In addition, these findings enhance our understanding as to why not all participants are experiencing benefits from the program. Second, we purposively sought a diverse representation of participants, including those in rural and urban areas as well as across payment models (e.g., paying privately or public coverage). This enables our results to better reflect the provincial context and be transferable to other regions with a similar implementation context (i.e., single-payer healthcare systems with limited coverage for the program). Future research may wish to explore patient experiences of the GLA:D program based on participants’ characteristics (e.g., race/ethnicity, diverse gender identity, income, age) or provider characteristics (e.g., type of provider) rather contextual factors that might require a larger sample size to achieve saturation. Despite these strengths, this study also has limitations that require consideration. First, the interviews occurred up to 12 months after the participants completed the program, which increases the potential for recall bias. While outside of the scope of the current project, future research might wish to examine whether the benefits reported in the present manuscript were sustained beyond 12 months. Second, member checking was not completed; therefore, the transcripts were not shared with patients for input and feedback, which increased the chance of researcher bias. Lastly, our research team did not include people living with OA; however, the overall evaluation of the GLA:D program was informed by patient advisors engaged in provincial OA initiatives.

## Conclusions

Individuals with hip and knee OA who participated in the GLA:D program perceived many benefits from participation including improvements in pain, joint function, strength, and mobility and they regained the ability to complete activities of daily living and participate in leisure and sports that they had previously given up. Participants also felt the program instilled a new sense of confidence and awareness allowing them to better manage their OA symptoms and ultimately resulting in motivation to embed the program learnings in their everyday lives to maintain the benefits. Participant experiences indicated that the program had an impact on their overall sense of well-being through regaining ease of movement, the relief that something helped their OA, the enjoyment of returning to activities, and not being restricted or limited in terms of their activities. For participants who did not experience benefits, this was largely due to pain, the severity of their OA, and the program’s failure to meet their anticipated goals. Earlier intervention with the GLA:D program and improved screening may assist in improving the number of participants who benefit from the program.

### Electronic supplementary material

Below is the link to the electronic supplementary material.

Supplementary Material 1

Supplementary Material 2

Supplementary Material 3

## Data Availability

The full data set is not publicly available for reasons of confidentiality. De-identified interview transcripts may be requested from the corresponding authors. Release of de-identified interview data will be dependent up on ethics review and approval.

## List of abbreviations

GLA:D: Good Life with osteoArthritis: Denmark
OA: Osteoarthritis
COREQ: Consolidated Criteria for Reporting Qualitative Research
