# Supplementary material for: Participants’ perceived benefits from the GLA:D™ program for individuals living with hip and knee osteoarthritis: a qualitative study
Source: J Patient Rep Outcomes. 2024 Jun 26;8:62. doi: 10.1186/s41687-024-00740-w (PMC11208368; doi:10.1186/s41687-024-00740-w)
Supplement: Supplementary file 2 — Supplementary Material 2 [file 41687_2024_740_MOESM2_ESM.docx]

**Additional File 2**: Semi-structured Interview Guide

1. Why did you sign up to take GLA:D?

a. What factors contributed to your decision to sign up to GLA:D?

2. *How did you come across the GLA:D program?

3. Have you done any other exercise programs for your OA?

a. What were they?

b. Where they similar to or different from GLA:D? How?

4. What was your experience of the GLA:D program? The education session? The exercise?

a. Did you find the exercise/education useful?

5. Do you think you benefited from doing GLA:D? how?

a. Would you do it again?

6. *Have you maintained the exercises after the GLA:D program?

7. What did you think of the cost of GLA:D?

8. *How much would you be willing to pay for GLA:D?

a. What other factors are important to the idea of “willingness to pay”

9. What would make it easier for you to take a program like GLA:D?

** Indicates questions added to the original semi-structured interview guide*
