# Supplementary material for: Participants’ perceived benefits from the GLA:D™ program for individuals living with hip and knee osteoarthritis: a qualitative study
Source: J Patient Rep Outcomes. 2024 Jun 26;8:62. doi: 10.1186/s41687-024-00740-w (PMC11208368; doi:10.1186/s41687-024-00740-w)
Supplement: Supplementary file 3 — Supplementary Material 3 [file 41687_2024_740_MOESM3_ESM.docx]

**Additional File 3:** Research Team Description and Reflexivity Statement

The research team consisted of two experienced non-clinician qualitative researchers AKR holds a PhD and EM holds an MSc, both in health services research. AKR was supported the operational implementation of GLAD in Alberta. DT was completing a Master’s degree during the project and had no prior knowledge of the GLA:D program. LAB and CAJ are senior academic researchers in bone and joint health, and experienced physical therapists. CAJ is actively involved in delivering GLA:D program clinical practice and provider GLA:D training courses. GJP is a doctoral candidate in public health. JR, MS, DAH and JM were involved in the implementation and evaluation of the GLA:D program, and members of the Bone and Joint Health Strategic Clinical Network leadership team. AKR, LAB, GJP, DT, JR, CAJ all identify as women, JM, DAH, MS identify as men. None of the team members had prior interactions with study participants and no information on interviewer characteristics or goals were explained to participants ahead of the interview.
